# Clinical Characteristics and Microbiology of Bloodstream Infections in Hemodialysis-Dependent Patients: A Multicenter Retrospective Study from Five Hemodialysis Centers in Saudi Arabia

**DOI:** 10.1055/s-0046-1824468

**Published:** 2026-06-16

**Authors:** Alaa T. Alsayed, Muhammad Nauman Hashmi, Qurat Ul Ain, Syed Raza, Fayez Hejaili

**Affiliations:** 1 Hemodialysis Care Project, Ministry of National Guard Health Affairs, Jeddah, Saudi Arabia; 2 Hemodialysis, King Abdullah International Medical Research Center, Jeddah, Saudi Arabia; 3 Hemodialysis Care Project, Ministry of National Guard Health Affairs, Riyadh, Saudi Arabia

**Keywords:** hemodialysis, bloodstream infection, catheter-related bloodstream infection, central venous catheter, vascular access, microbiology, infection

## Abstract

**Background:**

Patients receiving hemodialysis are at high risk for bloodstream infections (BSIs), particularly those with central venous catheters (CVCs). Regional variation in microbial patterns has been reported, with studies from Saudi Arabia suggesting a higher prevalence of gram-negative pathogens compared with Western cohorts. This study aimed to describe the clinical characteristics and microbiology of BSIs among hemodialysis-dependent patients treated within the Ministry of National Guard Health Affairs in Saudi Arabia and to compare findings with international data.

**Methods:**

We conducted a retrospective, multicenter study across five hemodialysis centers in Saudi Arabia over a 5-year period (January 2019–December 2023). Adult patients (≥15 years) on maintenance hemodialysis with confirmed BSI based on positive central or peripheral blood cultures were included. Demographic data (including age and body mass index [BMI]), comorbidities, vascular access type, microbiological isolates, and clinical outcomes were evaluated.

**Results:**

A total of 437 hemodialysis patients with positive blood cultures were identified, yielding 709 microbial isolates. The study population had a median age of 58 years, and obesity was prevalent (41.7% with BMI >30 kg/m ^2^ ). CVCs were present in 92.7% of BSI episodes, while arteriovenous fistulas or grafts accounted for 7.3%. Hypertension (88%) and diabetes mellitus (60%) were the most common comorbidities. Overall, gram-negative organisms predominated (51.8%), followed by gram-positive organisms (48.2%). *Staphylococcus aureus* was the most frequent gram-positive pathogen, while *Enterobacter cloacae* and *Klebsiella pneumoniae* were the leading gram-negative isolates. Catheter removal was performed in 57.3% of cases, while catheter salvage was successfully attempted in 42.7%. The 4-week mortality was remarkably low (1.14%).

**Conclusion:**

BSIs in hemodialysis patients are predominantly catheter-associated and demonstrate a substantial gram-negative burden, differing from traditional Western patterns. High obesity rates and prolonged CVC reliance underscore the need for region-specific prevention strategies, including early permanent access creation, strict catheter management, and antimicrobial stewardship. The favorable mortality rate highlights the effectiveness of current early detection and management protocols.

## Introduction

Hemodialysis is the predominant modality of kidney replacement therapy (KRT) globally, accounting for approximately 69% of all KRT and nearly 89% of all dialysis treatments. 1 As of 2023, the global prevalence of kidney failure requiring replacement therapy—including both dialysis and transplantation—was estimated at 4.59 million cases, with hemodialysis representing the majority of these patients. 1 2 Hemodialysis patients are at markedly increased risk for infections, with catheter-related bloodstream infections (CRBSIs) being the most common and clinically significant infectious complication. The risk is highest in those with central venous catheters (CVCs), especially non-tunneled catheters, but remains elevated for tunneled catheters and arteriovenous grafts (AVGs) compared with arteriovenous fistulas (AVFs). 3 4 Furthermore, patient-specific factors, including advancing age and rising obesity rates, complicate vascular access management and heighten the baseline risk for infection in this vulnerable population.

Bacteremia in hemodialysis patients is characterized by distinct microbial patterns, with both gram-positive and gram-negative organisms playing significant roles. Polymicrobial bacteremia occurs in a minority of cases but is clinically relevant. 5 The microbial spectrum is influenced by the type of vascular access, with CVCs conferring a higher risk for both gram-positive and gram-negative infections. 5 6 The incidence of bloodstream infection (BSI) in hemodialysis patients is substantially higher than in the general population, with rates ranging from 0.52 to 13.7 episodes per 1,000 patient-days or per 100 person-years, depending on the study and population. 7 8

Bacteremia in the hemodialysis population leads to significant morbidity, including prolonged hospitalizations (median 9 days), persistent bacteremia, metastatic complications (such as endocarditis and osteomyelitis), and septic shock. 5 8 Short-term mortality following bacteremia is high, with 30-day case fatality rates of 16% to 18%. 7 8 9 The majority of BSIs are related to vascular access, especially CVCs, but non-access sources (e.g., intra-abdominal, urinary, diabetic foot ulcers) are increasingly recognized and associated with higher mortality. 5 8 10

The pattern of BSIs among patients undergoing dialysis in Saudi Arabia is characterized by a relatively high incidence of access-associated bacteremia, with a notable predominance of gram-negative organisms compared with international benchmarks. Prospective surveillance at major Saudi centers has demonstrated that rates of access-associated bacteremia are two to four times higher than those reported in the U.S. National Healthcare Safety Network, with a higher prevalence of CVCs contributing to this increased risk. Gram-negative rods account for approximately 48% of positive blood cultures in Saudi hemodialysis outpatients, which is significantly higher than the 21% observed in the U.S. cohorts. Gram-positive organisms, particularly Staphylococci, remain important, but the proportion of gram-negative pathogens is distinctly elevated in the Saudi population. 11 12 These findings underscore the importance of infection control measures, judicious vascular access selection, and ongoing surveillance of antimicrobial resistance in Saudi hemodialysis populations.

However, there is a lack of recent multicenter data that integrate clinical outcomes—such as mortality and catheter salvage success—with these shifting microbial patterns in the region. Our study aimed to identify pathogens in BSIs of hemodialysis patients dialyzing in centers under the Ministry of National Guard Health Affairs (MNGHA) and to compare them with international and local statistics. By evaluating the microbial spectrum alongside demographic and clinical outcomes, we aim to provide an evidence base for implementing targeted infection control bundles and antimicrobial stewardship programs to reduce BSI rates and improve patient survival.

## Methods

A retrospective study of positive blood cultures was conducted in adult patients receiving hemodialysis in five centers operated under the Ministry of National Guard Health Affairs (MNGHA; Jeddah, Makkah, Madinah, and two centers in Riyadh). Study approval was granted by the King Abdullah International Medical Research Centre (KAIMRC) and the Institutional Review Board.

Data were extracted from the electronic health record system (BestCare) over a 60-month period (January 2019–December 2023). Eligible participants included adult patients (aged ≥15 years) on maintenance hemodialysis who developed BSIs. BSI was defined by the isolation of a pathogen from at least one set of peripheral or central blood culture. Patients with temporary catheters, those on peritoneal dialysis, transient (tourist) patients, and those with incomplete records were excluded. Furthermore, contaminated samples were strictly excluded based on the Centers for Disease Control and Prevention's National Healthcare Safety Network (CDC/NHSN) criteria, 13 defined as skin commensals (e.g., coagulase-negative Staphylococci) isolated in only one blood culture set in the absence of clinical signs of sepsis.

Information on patient characteristics, hemodialysis history, vascular access details, and positive blood culture information was collected and documented in a secure database. Access was restricted to the principal investigator and co-investigators to ensure data confidentiality. Variables evaluated included demographics (age and body mass index [BMI]), comorbidities (e.g., hypertension, diabetes), vascular access type (CVC, AVF, or AVG), and clinical outcomes (including catheter removal, salvage, and 4-week mortality).

Data analysis was conducted using SPSS Statistics for Windows, version 26.0 (SPSS Inc., Chicago, IL, United States). Descriptive statistics summarized patient demographics, clinical characteristics, and episodes of bacteremia. Categorical variables were expressed as frequencies and percentages, while continuous variables were reported as means with standard deviations or medians with interquartile ranges (IQRs), as appropriate.

## Results

### Patient Demographics and Clinical Characteristics

**Fig. 1:**
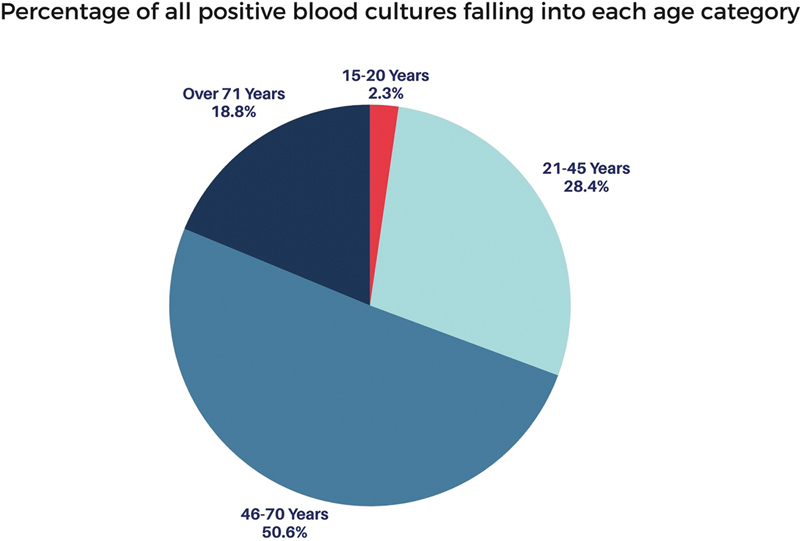
Distribution of total bloodstream infections by patient age group ( *N* = 437).

The study included 437 hemodialysis patients with positive blood cultures. The cohort exhibited a median age of 58 years (IQR: 46–70 years), with 61.0% of patients being male. The majority of positive blood cultures occurred in patients aged 40 years and older (50.6%); age distribution is further detailed in Fig. 1 . In terms of BMI, obesity was prevalent, with 41.7% ( *n* = 182) of patients having a BMI of >30 kg/m ^2^ , while 32.1% ( *n* = 140) were classified as overweight (BMI 25–30 kg/m ^2^ ). CVCs were the predominant vascular access type, present in 92.7% of BSI episodes, whereas permanent access (AVF/AVG) accounted for only 7.3%. Comorbidity profiles revealed high rates of hypertension (88%) and diabetes mellitus (60%), with 18% of patients having ischemic heart disease. Notably, the distribution of BSI episodes correlated with BMI categories, where patients with a BMI >30 accounted for 41.7% of cases, reflecting the potential technical challenges in accessing care within this subgroup.

### Microbial Profile

A total of **709** microbial pathogens' isolates were identified from central and peripheral blood culture samples. Gram-negative organisms slightly surpassed gram-positive isolates in the overall distribution. Table 1 summarizes the classification of these pathogens.

**Table 1:** Distribution of microbial isolates ( *N* = 709)

Classification	Central samples ( *N* = 386)	Peripheral samples ( *N* = 323)	Total isolates ( *N* = 709)	*p* -Value
Gram positive	169 (43.8%)	158 (48.9%)	**327 (46.1%)**	**0.17**
Gram negative	200 (51.8%)	150 (46.4%)	**350 (49.4%)**	**0.15**
Other (fungi/mixed)	17 (4.4%)	15 (4.6%)	**32 (4.5%)**	**0.89**

Regarding specific pathogens, *Staphylococcus aureus* (methicillin-sensitive *S. aureus* [MSSA]) was the most frequent single pathogen isolate (21.2% central, 21.4% peripheral), followed by *Enterobacter cloacae* (11.7%) and *Klebsiella pneumoniae* (10.4%). Resistance patterns were clinically significant: 24.1% ( *n* = 26/108) of the *S. aureus* isolates were methicillin-resistant (MRSA). Furthermore, a significant proportion of gram-negative isolates exhibited multidrug-resistant organism (MDRO) characteristics, particularly resistance to third-generation cephalosporins, which aligns with the high “antibiotic pressure” noted in the study environment.

To identify independent risk factors for BSI, a multivariable logistic regression analysis was performed ( Table 2 ). CVC use was identified as the strongest independent predictor of BSI (adjusted odds ratio [OR]: 12.4, 95% confidence interval [CI]: 8.2–18.7, *p* < 0.001), indicating a 12-fold increased risk compared with permanent access. While advanced age (>65 years) and diabetes showed a trend toward increased risk, they did not reach statistical significance in this model.

**Table 2:** Multivariable logistic regression analysis of risk factors for bloodstream infection ( *N* = 437)

Risk factor	Adjusted odds ratio	95% Confidence interval	*p* -Value
CVC use (vs. permanent access)	12.4	8.2–18.7	<0.001 a
Diabetes mellitus	1.35	0.92–1.98	0.12
Age (>65 years)	1.12	0.85–1.48	0.41
Gender (female)	0.98	0.71–1.35	0.89

Abbreviation: CVC, central venous catheter.

a *p* -Value <0.05 is considered statistically significant.

### Clinical Outcomes and Mortality

Among BSI episodes related to CVCs ( *n* = 405), catheter removal was performed in 57.3% ( *n* = 232) of cases, whereas catheter salvage was successfully attempted in 42.7% ( *n* = 173). A total of 48 cases (11.0%) were attributed to non-access source (e.g., intra-abdominal or skin infections). The overall 4-week mortality rate following the diagnosis of BSI was remarkably low at 1.14% ( *n* = 5). All fatalities occurred among patients with multiple comorbidities dialyzing via CVCs.

## Discussion

BSIs are an important cause of hospitalizations, morbidity, and mortality in patients receiving hemodialysis. Eliminating BSIs in the hemodialysis setting has been the focus of the Centers for Disease Control and Prevention Making Dialysis Safer for Patients Coalition and, more recently, the CDC's partnership with the American Society of Nephrology's Nephrologists Transforming Dialysis Safety Initiative. 14

Regarding the demographic profile, the study population had a median age of 58 years, and a high prevalence of obesity (41.7%) was noted. These demographic characteristics are clinically significant, as obesity technically challenges the maintenance of aseptic vascular access sites and the creation of permanent access, potentially contributing to the prolonged reliance observed in our cohort.

The risk of BSIs in hemodialysis is strongly influenced by the type of vascular access. CVCs are associated with the highest risk of BSIs, followed by AVGs, with AVFs carrying the lowest risk. In a systematic review of over 580,000 patients, tunneled CVCs were associated with a 1.5- to 2-fold higher risk of infection compared with arteriovenous access (AVF or AVG). 3 To address the high CVC dependency (92.7%), our centers utilize specialized multidisciplinary “vascular access clinics” and dedicated coordinators to initiate permanent access planning as early as stage 4 chronic kidney disease (CKD). These resources are essential to overcome regional barriers such as late nephrology referral and patient reluctance, which remain significant challenges in our clinical context.

The microbiology of BSIs in hemodialysis patients is characterized by a predominance of *S. aureus* (including MRSA), coagulase-negative *Staphylococcus* , and an important contribution from gram-negative organisms, such as *Escherichia coli* , *Serratia marcescens* , and *Pseudomonas aeruginosa* . MDROs are a major concern, with a high proportion of both gram-positive and gram-negative isolates demonstrating resistance to multiple antibiotic classes. 5 15

In our 5-year results, gram-positive organisms, along with gram-negative organisms, share nearly equal burden as causative organisms for BSI, with a slight predominance of gram-negative pathogens. This shift, where gram-negative bacilli (GNB) accounted for 51.8% of isolates in our study, is a critical finding that distinguishes the microbial landscape in Saudi Arabia from the traditional gram-positive predominance reported in international literature, providing new evidence base for local antimicrobial stewardship. This regional GNB predominance necessitates that local antimicrobial protocols be updated to ensure adequate early coverage for these pathogens. This regional GNB predominance is likely exacerbated by “antibiotic pressure”—the selective evolutionary pressure where frequent use of broad-spectrum antimicrobials eliminates sensitive flora, allowing resistant gram-negative strains to emerge as dominant pathogens in the dialysis settings. Our findings regarding antibiotic pressure are consistent with global observations that intensive antimicrobial exposure in dialysis units correlates with a higher prevalence of resistant gram-negative bacteria. 10 16 17 The proportion of gram-negative versus gram-positive organisms varies significantly by region, and this variability is associated with latitude, climate, and socioeconomic determinants. Centers closer to the equator and those with lower health care expenditure tend to have a higher fraction of gram-negative bacteremia, likely due to climatic factors favoring gram-negative bacterial proliferation and differences in infection control infrastructure. 18 Geographic disparities in the microbial patterns causing bacteremia in hemodialysis patients are multifactorial and reflect both environmental and health system factors.

Socioeconomic factors, including the proportion of gross domestic product spent on health care, are independently associated with microbial patterns. Regions with lower health care investment may have less robust infection prevention protocols, suboptimal vascular access management, and higher rates of antimicrobial resistance, all of which influence the spectrum of pathogens encountered. 16 18 Excessive and inappropriate antibiotic use, as observed in some settings, drives colonization and infection with resistant gram-negative organisms, and the diversity of clones suggests multiple sources of transmission within dialysis centers. 16

While environmental factors such as local water quality are occasionally discussed, our data suggest that it is not a primary risk factor for CRBSIs in this cohort. Instead, the observed infections primarily reflected pathogens associated with catheter manipulation and “antibiotic pressure,” rather than external environmental contaminants. Furthermore, our clinical management included a catheter salvage rate of 42.7%, achieved through strict protocols, systemic antibiotics, and antibiotic lock therapy. The effectiveness of these interventions, combined with early detection, is reflected in the remarkably low 4-week mortality rate of 1.14% observed in our study, which is notably lower than many international cohorts where mortality can range from 10% to 20%. 7 19 Finally, disparities in vascular access practices and social determinants of health, such as poverty, crowding, and education, further modulate infection risk and microbial patterns. For example, higher rates of CVC use in resource-limited settings are associated with increased risk of *S. aureus* bacteremia, while environmental and antibiotic pressure may favor gram-negative pathogens. 16 20 By addressing regional barriers through integrated strategies—ranging from stage 4 CKD planning to specialized nursing care—we can further reduce the infection burden and improve outcomes in our regional clinical context.

**Fig. 2:**
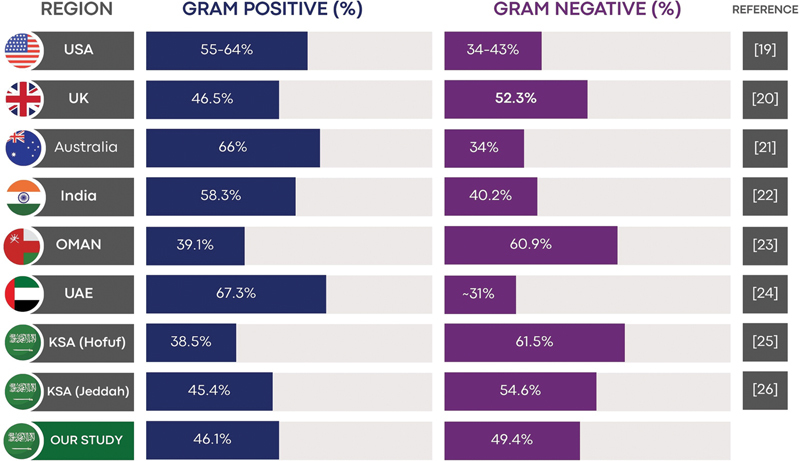
Comparison of gram-positive and gram-negative bacteria, globally versus our study.

Fig. 2 summarizes studies across different regions, 19 21 22 23 24 25 26 27 and a comparison with our study. There is a wide difference in the spectrum of microbes. As we discussed earlier, the following are the key contributing factors for this variation.

- Vascular access differences
- Antibiotic use and resistance
- Infection control practices
- Patient demographics
- Climate of the region
- Local microbiological ecology

**Fig. 3:**
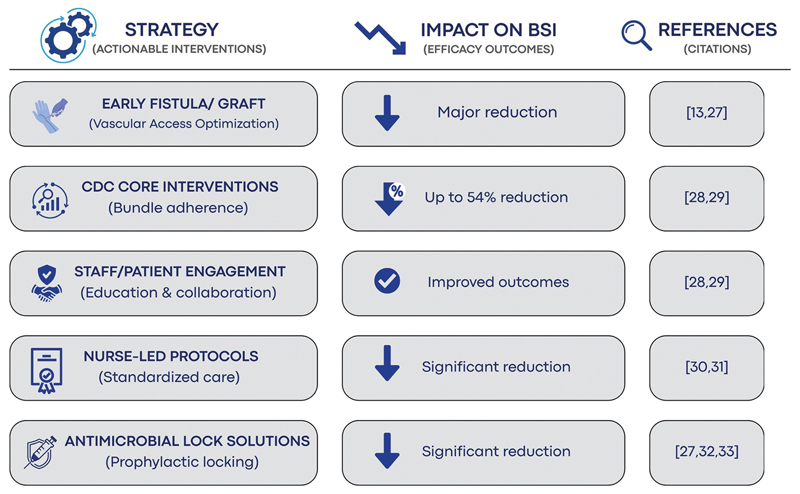
Key strategies for reducing bloodstream infection: Evidence-based take-home messages.

The key strategies for reducing BSIs, as illustrated in Fig. 3 , rely on a multifaceted approach. By optimizing vascular access through dedicated clinics and adhering to CDC core interventions, 28 29 this significantly mitigates infection risks. Furthermore, the integration of staff and patient education, 28 29 nurse-led protocols, 30 31 and antimicrobial lock solutions 32 33 provides a robust framework for sustained BSI reduction in the centers.

### Study Limitations

The study has several limitations that should be acknowledged. First, its retrospective nature may have resulted in missing data for some secondary clinical variables. Second, while the study included five major centers, the results primarily reflect the microbial patterns and clinical practices within a specific geographical region of Saudi Arabia, which may limit the generalizability to different international healthcare settings. Finally, the study focused on clinical and microbiological outcomes without assessing the long-term mortality beyond the hospital stay. Despite these limitations, the large sample size and multicenter design provide robust evidence regarding the current burden of BSIs in our dialysis population.

## Conclusion

Global variation in microbial patterns among hemodialysis patients is driven by a complex interplay of vascular access types, regional antibiotic practices, infection control standards, and local microbial ecology. Our study highlights a significant shift toward gram-negative pathogens and a high prevalence of obesity, which technically challenges vascular access care and prolongs catheter dependency. Despite these challenges, strict clinical protocols and effective salvage strategies achieved favorable outcomes with low mortality. These findings necessitate tailored antimicrobial stewardship and continued emphasis on early permanent access. Implementing these integrated strategies is essential to reducing the infection burden and improving outcomes within our regional clinical context.
